# Self-organized alternating chimera states in oscillatory media

**DOI:** 10.1038/srep09883

**Published:** 2015-04-30

**Authors:** Sindre W. Haugland, Lennart Schmidt, Katharina Krischer

**Affiliations:** 1 Physik-Department, Nonequilibrium Chemical Physics, Technische Universität München, James-Franck-Str. 1, D-85748 Garching, Germany; 2 Institute for Advanced Study – Technische Universität München, Lichtenbergstr. 2a, D-85748 Garching, Germany

## Abstract

Oscillatory media can exhibit the coexistence of synchronized and desynchronized regions, so-called chimera states, for uniform parameters and symmetrical coupling. In a phase-balanced chimera state, where the totals of synchronized and desynchronized regions, respectively, are of the same size, the symmetry of the system predicts that interchanging both phases still gives a solution to the underlying equations. We observe this kind of interchange as a self-emerging phenomenon in an oscillatory medium with nonlinear global coupling. An interplay between local and global couplings renders the formation of these alternating chimeras possible.

Synchronization phenomena are omnipresent in nature, and, consequently, their theoretical description has received great attention for the last two decades^1^. Given a network of coupled oscillators with a distribution of natural frequencies, it was a seminal achievement to describe their synchronization transition when the coupling strength is increased^2^. Such a transition can be observed in populations of flashing fireflies, in a clapping audience, in coupled pendulum clocks or metronomes and in many other natural systems^3^. Lately, a contrasting and more counterintuitive transition has received considerable interest from the nonlinear dynamics community: a population of identical oscillators with symmetrical coupling can split into two coexisting groups, one oscillating in synchrony, while the other one behaves desynchronized. These so-called chimera states have been the subject of several theoretical studies^4^,^5^,^6^,^7^,^8^,^9^,^10^,^11^,^12^,^13^,^14^,^15^, and could be realized in a number of different experimental systems^12^,^14^,^16^,^17^,^18^,^19^; for a review, see Ref. 20.

The possible importance of chimera states ranges across various disciplines, pertaining to phenomena such as the unihemispheric sleep of animals^21^,^22^,^23^, signal propagation through synchronized firing in otherwise chaotic neuronal networks^24^, and the existence of turbulent-laminar patterns in Couette flow^25^. So far, chimera states typically show a persistent separation into coherent and incoherent domains, with no interchange of dynamics between the different domains. During unihemispheric sleep, however, when one half of the brain stays awake and shows desynchronized neuronal activity while the other half is synchronized and sleeping, the synchronization of neurons is known to alternate between cerebral hemispheres^21^,^22^,^23^. In theoretical studies this phenomenon could only be reproduced by considering two man-made groups of non-identical oscillators with predefined inter- and intra-group coupling, either autonomously^26^ or driven by a periodic external signal^27^.

In contrast, in this Article we present alternating chimera states that spontaneously emerge in an isotropic oscillatory medium with nonlinear uniform global coupling, thereby tightening the connection between chimera states and unihemispheric sleep. As the chimera states found so far in this system are in phase balance, and since the parameters are uniform and the coupling is symmetric, interchanging the incoherent and coherent phases again yields a solution of the underlying equations. A combination of local and global coupling effects then triggers the alternation.

## Results

The model we consider is a spatially two-dimensional system governed by a modified complex Ginzburg-Landau equation (MCGLE)^14^,^28^:
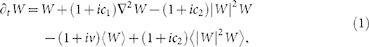
where a linear and a nonlinear global coupling term have been added to the standard complex Ginzburg-Landau equation (CGLE). Here *W* = *W*(*x, y, t*) is a complex variable describing the dynamical state of the system at any location (*x, y*) at time *t* and 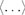 denotes spatial averages. By spatially averaging the MCGLE, one obtains a rather simple equation for the dynamics of 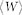:
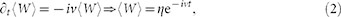
displaying simple harmonic oscillations with amplitude *η* and angular frequency *v*. The standard CGLE is a generic model for a spatially extended system close to the onset of oscillations, and is considered one of the most important nonlinear equations in physics^29^,^30^. To account for peculiar pattern formation in the oxide-layer thickness observed during the photoelectrodissolution of n-type silicon^14^,^31^,^32^, we introduced the nonlinear global coupling. For a wide range of experimental parameters and various types of spatial patterns, the spatially averaged oxide-layer thickness has been found to display nearly harmonic oscillations^31^,^32^. This is captured with the special nonlinear global coupling in Eq. (1), as shown in Eq. (2).

**Figure 1:**
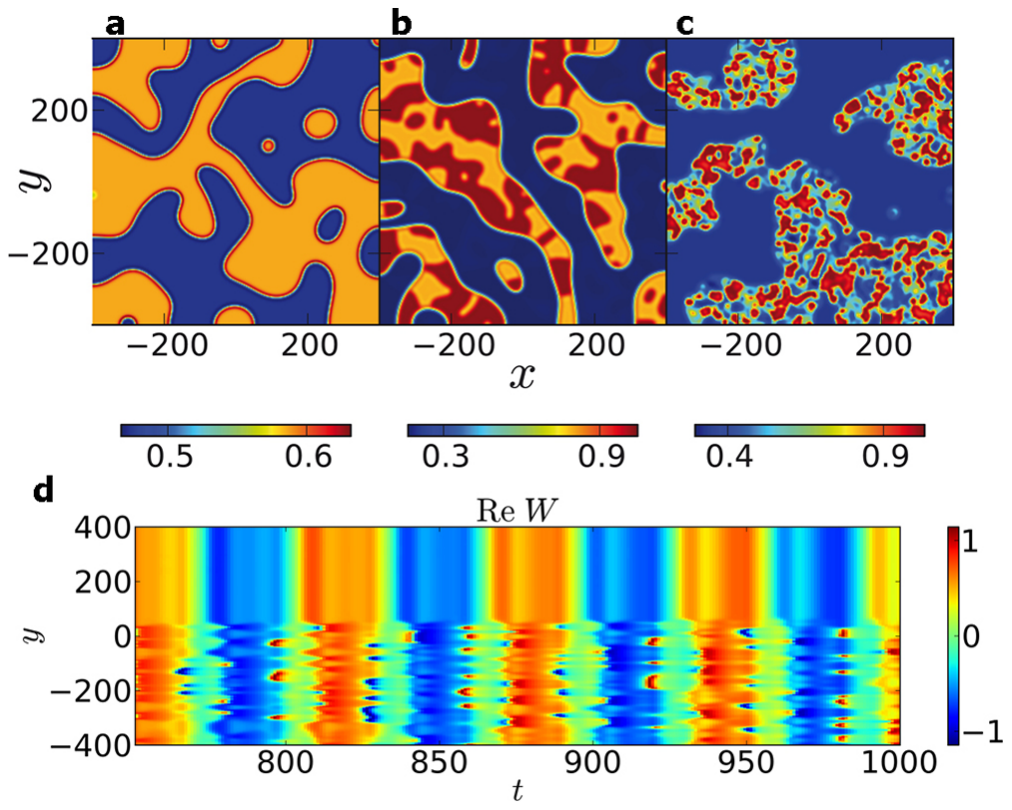
Transition from two-phase clusters to a two-dimensional chimera state. Shown are snapshots of the real part of the complex variable *W* in (a)–(c). (a) Two-phase clusters obtained for parameter *c*_2_ = −0.7. Both phases are homogeneous. (b) Subclustering at *c*_2_ = −0.67. In this case, one phase is homogeneous, while the other one is split into two-phase clusters. (c) Two-dimensional chimera state found for *c*_2_ = −0.58. The inhomogeneous phase shows strongly incoherent dynamics. (d) Temporal evolution of the real part of *W* in a one-dimensional cut at *x* = 0 in (c). Other parameters read: *c*_1_ = 0.2, *v* = 0.1 and *η* = 0.66. Reprinted with permission from L. Schmidt, K. Schönleber, K. Krischer & V. García-Morales, *Chaos* 24, 013102 (2014). Copyright 2014, AIP Publishing LLC.

Numerically solving Eq. (1) for appropriate simulation parameters *c*_1_, *c*_2_, *v* and *η* (see methods section for details), we were able to reproduce different kinds of dynamics observed in the experimental silicon system, including a two-dimensional chimera state^14^: By changing the parameter *c*_2_ we observe a transition from two-phase clusters (Fig. 1a) to subclustering (Fig. 1b), where one of the two phases exhibits again two-phase clusters as a substructure. By further changing *c*_2_ we find a two-dimensional chimera state as shown in Fig. 1c; the spatio-temporal dynamics are visualized in a one-dimensional cut along *y* in Fig. 1d.

The chimera states previously obtained with the MCGLE exhibit a persistent division of the system into one coherent and one turbulent phase, each of which consists of one or more domains. After their initial formation, almost no area is exchanged between the phases.

**Figure 2:**
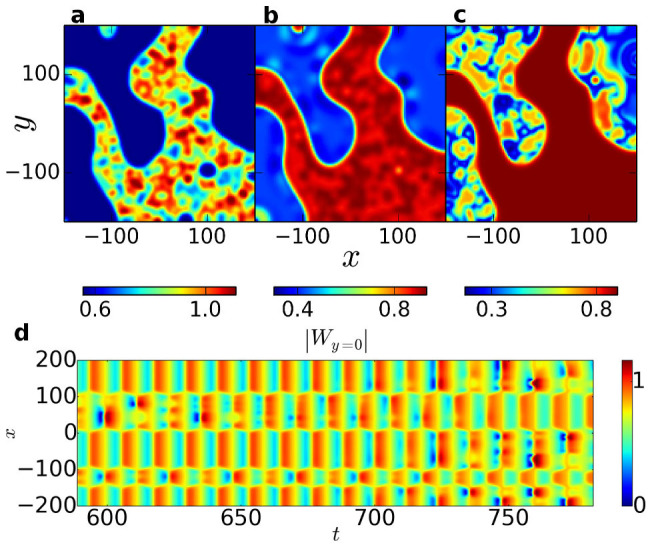
Alternating chimera state in the MCGLE. (a) Coexistence of turbulence and synchrony. (b) Spread of turbulence to the initially synchronized phase. (c) After an interval Δ*t* ≈ 200, the turbulence has moved completely from one phase to the other. (d) Temporal evolution of the absolute value of ω in a cross-section along the x-axis, covering the time interval from (a) to (c).

**Figure 3:**
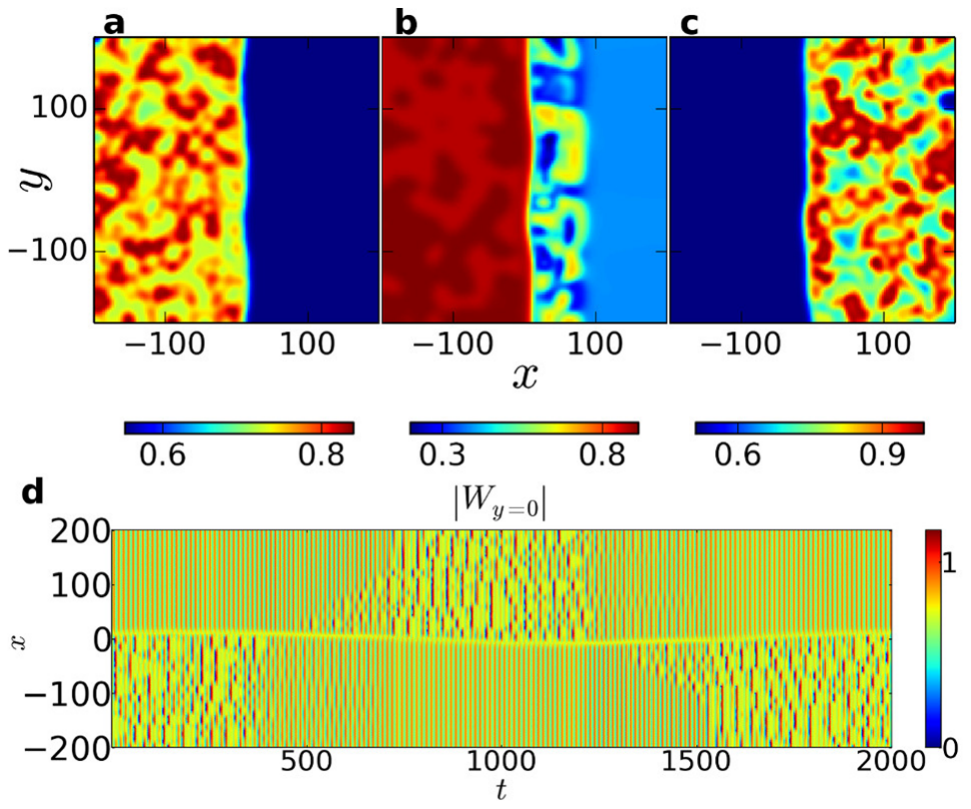
Alternating chimera state with straight boundary. (a–c) Three snapshots of the system after the two-domain state has been reached, recorded at relative points in time t = 0, 600 and 1000 Over the course of an interval Δ*t* ≈ 10^3^, the turbulence moves completely from one phase (a) to the other (c). (d) Temporal evolution of the absolute value of *W* in a cross-section along the *x*-axis. Alternations are now observed regularly at intervals of about Δ*t* ≈ 10^3^.

Changing *c*_2_ to *c*_2_ = −0.64, we observe an astonishing, new kind of chimera state. The initial transition to a state of one synchronized and one turbulent phase proceeds as for the “ordinary” chimera states. However, after some time interval the dynamics in the phases interchange, the initially synchronized phase becoming turbulent while the turbulent phase becomes synchronized, as depicted in Fig. 2. Initially, these alternations do not have a characteristic timescale, but occur rather erratically in time. Domains of the same phase tend to merge, thereby reducing curvature and length of the boundary between the phases. Eventually (at *t* = 10^6^), only two domains are left, separated by a roughly straight boundary along one of the axes of the system. Three snapshots in Figs. 3a–c visualize this situation. Now the alternations of turbulence and synchrony occur more regularly than before the two phases have properly demixed, approximately once every interval Δ*t* = 10^3^. We find these approximately regular alternations to persist for at least *t* = 3.7·10^7^, our maximum simulation time. Moreover, the system was simulated with the same parameter values for 100 slightly differing random initial conditions, always eventually leading to an alternation, the latest occurring at *t* ≈ 6·10^4^. In contrast, the system was simulated with the parameter values corresponding to the non-alternating chimera state in Fig. 1c for *t* = 10^6^ without an alternation taking place.

The two-domain state facilitates the study of the alternation process, which always proceeds similarly: First, the turbulence in the unsynchronized domain turns into a two-phase subclustering. Then a spatial pattern emerges in the previously coherent domain, starting at the domain boundary, which acts as a nucleus for turbulence (cf. Figs. 2b and 3b). As the incoherence spreads throughout the whole domain, the pattern in the other, originally turbulent domain gradually fades away, leaving it fully synchronized. This can be seen in Fig. 3d, where the temporal development of a cross-section is shown.

After the turbulence has engaged the formerly synchronized domain, the now turbulent domain grows further in size. This growth process is much slower than the preceding spread of turbulence within the domain. Eventually, the turbulent domain becomes larger than the synchronized one and when a critical size is reached, another alternation takes place.

**Figure 4:**
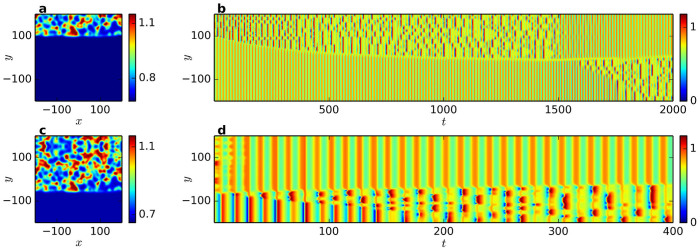
Modified initial states not satisfying phase balance. (a) Initially the turbulent domain is much smaller than the synchronized domain. (c) Here, the turbulent domain is initially larger. (b,d) Temporal evolution of cross-sections through the system evolving from (a) and (c), respectively.

In order to validate the above proposed mechanism, we used initial conditions as shown in Figs. 4a and c, where either the turbulent or the synchronized domain was chosen significantly larger than the other one. Starting out with turbulence covering only a small part of the system (Fig. 4a), this domain simply grows steadily until it covers slightly more than half the system, followed by an interchange of dynamics between the domains. This behavior is visualized in a one-dimensional cut shown in Fig. 4b. Notably, the growth rate is found to be greatest at the beginning and to gradually decrease when approaching phase balance. This can be rationalized as follows: Many cluster states, including the chimera state found in the MCGLE, display phase balance. Thus, the phase balanced state, where both phases cover the same area, is a preferred state, in the sense that the ghost of the stable phase-balanced state is still felt. The more the system approaches the phase balanced state, the slower the dynamics become.

When starting the simulation from a state where most of the system is covered by turbulence (Fig. 4c), an initial, very rapid synchronization takes place, with the originally turbulent domain becoming homogeneous within Δ*t* = 40. This rapid synchronization is followed by a spread of turbulence throughout the initially synchronized domain, and the same kind of steady domain growth as observed for the simulation where the turbulent domain is initially smaller. This corroborates that if the turbulent domain becomes too large, an alternation is triggered. Note that the growth rate of the turbulent nucleus in Fig. 4d is not symmetric in positive and negative *y*-direction. This could be a manifestation of the growth being governed by two different mechanisms. The growth process in the negative *y*-direction is the spreading of turbulence within the initially homogeneous domain. In the positive *y*-direction, the domain boundary is moving, at a slower pace.

For parameters corresponding to a non-alternating chimera state, initializing the system out of phase balance leads to the following behavior: an initially smaller turbulent domain grows until phase balance is reached, while for an initially larger turbulent domain, an alternation takes place at first, followed by the growth of the new, smaller turbulent domain up to phase balance. Thus, the difference between alternating and non-alternating chimeras is that in the former the phase balanced state is not stable and the turbulent domain grows further.

**Figure 5:**
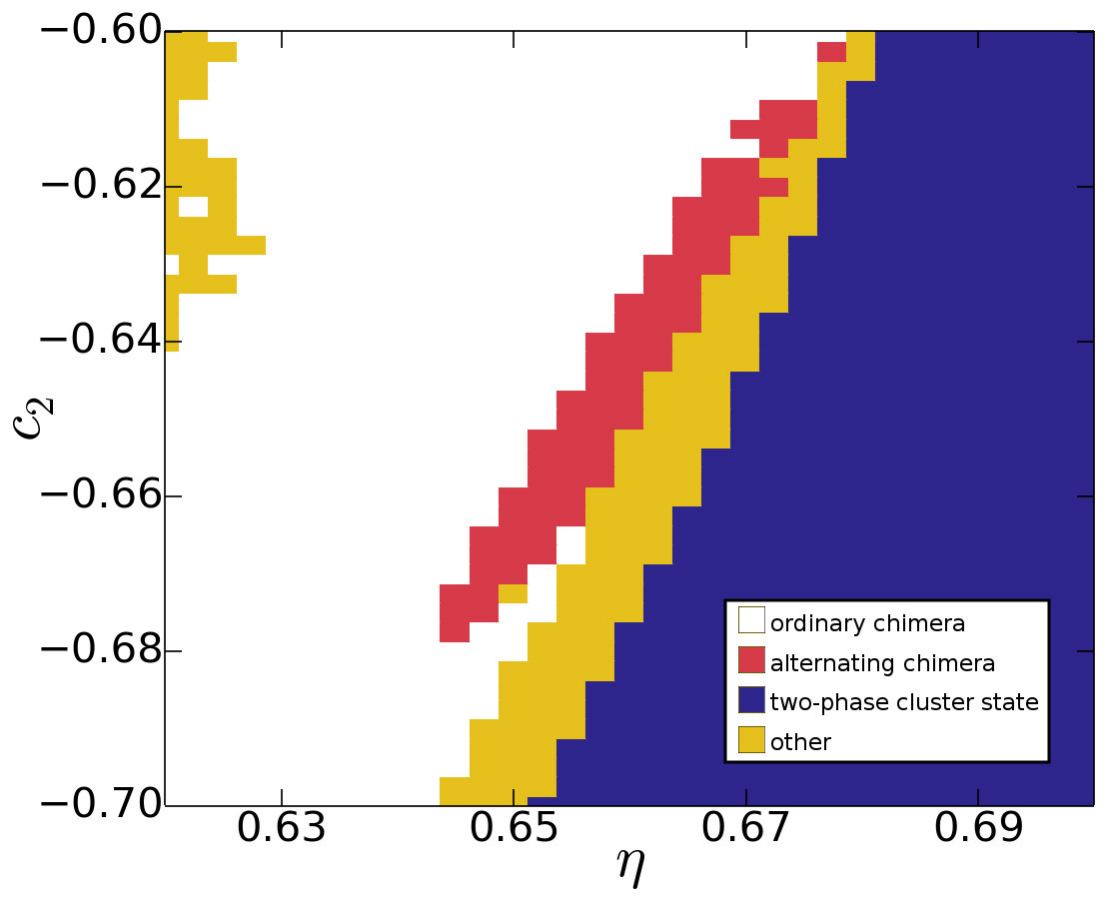
Phase diagram in the *c*_2_ vs. *η* parameter space containing regions of ordinary chimera states (white), alternating chimera states (red), two-phase cluster states (blue), and other dynamics (yellow), respectively. The latter group encompasses various different types of dynamics, including two-phase cluster states with subclustering, turbulence/two-phase subclustering combinations and two-phase states with turbulence in both phases.

Investigating the extent of alternating chimera states in parameter space yields the phase diagram depicted in Fig. 5. As shown, they span a distinct band-like region of the *η* – *c*_2_ parameter space, forming a part of the border between ordinary chimera states and two-phase cluster states.

Moreover, when carrying out simulations for *c*_2_ = −0.66 and *η* = 0.66, closer to the parameter range where two-phase subclustering was detected previously^14^, the alternation of the subclustering from one of the phases to the other is repeatedly found as an initial transient. However, in contrast to the alternating chimeras, this behavior has not yet been found to persist as long-term behavior.

### Conclusion

In summary, our simulations of a two-dimensional oscillatory medium governed by a complex Ginzburg-Landau equation with additional nonlinear global coupling, give evidence that alternating chimera states may spontaneously occur in isotropic oscillatory media. The simulations suggest that alternations are the result of an interplay between a diffusion-driven expansion of the turbulent phase and a global restriction on its maximum size, as demonstrated in Fig. 4. Moreover, movement of the boundary between the phases was found to always proceed in the direction of expansion of the turbulent phase. This is in accordance with earlier work on reaction-diffusion systems^33^,^34^,^35^,^36^,^37^, including the realistic model of catalytic CO oxidation on a Pt(110) surface^33^,^34^,^37^, where the expansion of turbulence at the expense of synchronized domains was observed as well.

The alternating behavior reminds of heteroclinic cycling between two attractors, similar to “slow switching” reported for two-cluster states in Refs. 38,39. However, at this state it would be premature to draw a conclusion about the mechanism of alternation.

As unihemispheric sleep of animals is suggested to be a prominent example of chimera states emerging in biological systems^21^,^22^,^23^, it is very important that the interchange of synchronization and incoherence between hemispheres occurring during this kind of sleep can be reproduced without external forcing and with identical oscillators. However, the current relation of chimera states and unihemispheric sleep has only a qualitative basis. Thus, the important next step in that direction would be the detailed investigation of neuronal dynamics during this sleep. Future research has to give answers to questions like what are appropriate models for neuronal oscillations and, even more importantly, how they are coupled.

## Methods

Simulations of Eq. (1) in the main text were carried out using a pseudospectral method, an exponential time stepping algorithm^40^ and a computational timestep of Δ*t* = 0.05. We used 256 × 256 Fourier modes, a system size of *L* = 400 and no-flux boundary conditions. Note that the equation is dimensionless.

All simulations except those shown in Fig. 4 were carried out from uniform initial conditions with superposed noise of 0.2%. Modified initial states not satisfying phase balance (Figs. 4a and c) were created by reflecting a chimera solution about *y* = ±200 (for a larger or smaller turbulent domain, respectively) and choosing an appropriate section of the enlarged system.

Simulation parameters *c*_1_ = 0.2, *v* = 0.1 and *η* = 0.66 were kept fixed for all simulations depicted in Figs. 1–4, while *c*_2_ was varied as described in the main text and caption of Fig. 1. When investigating the extent of alternating chimera states in parameter space, *c*_1_ = 0.2 and *v* = 0.1 were still left constant, while *η* and *c*_2_ were varied as shown in Fig. 5.

In order to classify the dynamics for a particular set of parameter values in the phase diagram in Fig. 5, simulations were initialized from a phase balanced state consisting of a homogeneous and a spatially turbulent domain, separated by a vertical boundary. If the turbulence switched twice from one side to the other within less than *t* = 2·10^4^, the dynamics were classified as an alternating chimera state. If the turbulent half remained turbulent throughout the pre-set time interval, while the synchronized half remained synchronized, or if they switched just once, the dynamics were classified as an ordinary chimera state. Two-phase cluster states were also classified correspondingly, while all other dynamics were combined into a fourth group of other dynamics (see Fig. 5).
